# Factors Contributing to Declining Swallowing Function in Older Adults and the Effectiveness of Self-Care Using Non-invasive Press Needles

**DOI:** 10.7759/cureus.79333

**Published:** 2025-02-19

**Authors:** Sumire Ishiyama, Satoshi Ayuzawa, Naomi Kuramoto

**Affiliations:** 1 Pain Management, Center of Medical Sciences, Ibaraki Prefectural University of Health Sciences, Ami, JPN; 2 Neurosurgery, Faculty of Health Sciences, Tsukuba University of Technology, Tsukuba, JPN; 3 Statistics, Center of Humanities and Sciences, Ibaraki Prefectural University of Health Sciences, Ami, JPN

**Keywords:** acupuncture patch, acupuncture point, acupuncture therapy, frail older adults, non-invasive press needle, self-care, swallow, swallowing function, swallowing sound, tongue pressure

## Abstract

Objective: Aspiration, especially silent aspiration associated with dysphagia, is a major cause of pneumonia. This is a disease that commonly occurs in the elderly, but the characteristics of the elderly who currently have no subjective swallowing problems are unclear. This study aims to identify factors contributing to declining swallowing function in elderly individuals and assess the effectiveness of non-invasive press needles as a self-care intervention.

Material and method: Our study is a prospective study. Twenty-seven individuals aged 65 years or over and without cause dysphagia were enrolled at the Tsukuba University of Technology, Japan. Twenty-eight individuals from 20 to 64 years of age were included in the younger age group. The amount of oral water content and tongue pressure were measured for all participants, and the repetitive saliva swallowing test (RSST) was performed using a Swallowscope. For individuals with reduced RSST swallowing function, non-invasive press needles were applied bilaterally to ST36 and KI3 acupuncture points and reassessed after one week.

Results: The average tongue pressure was significantly lower in the older adult group. In RSST, declining swallowing functions were observed in seven participants in the older adult group and four in the younger age group. With self-care interventions using press needles, average RSSTs significantly improved in the older adult group from 1.4±0.8 to 2.9±1.3 (p<0.05). In four participants with declining swallowing ability in the younger age group, the swallowing frequency remained less than three after the intervention.

Conclusion: The swallowing function potentially declines in older adults. Non-invasive press needles can be utilized to prevent dysphagia as a self-care.

## Introduction

Pneumonia is the fifth leading cause of death in Japan, occurring most frequently in elderly individuals aged over 65 years [1]. Aspiration, especially silent aspiration associated with dysphagia, is a major cause of pneumonia. It has been pointed out that even among elderly people who do not present with clinical dysphagia, there is a subjective and objective decline in swallowing function [2,3]. It is important to prevent aspiration pneumonia by detecting a decline in swallowing function in the elderly at an early stage and maintaining and improving swallowing function by providing early intervention.

The gold standard for evaluating swallowing function in clinical settings is videofluorography (VF) and videoendoscopic evaluation of swallowing (VEES); however, these involve invasive procedures, such as exposure to radiation. In contrast, the repetitive saliva swallowing test (RSST) is a relatively easy and non-invasive method. The RSST can be performed at the bedside, and it has been reported that the sensitivity and specificity of the test for identifying cases of aspiration are 0.98 and 0.66, respectively, making it an established method for assessing impaired swallowing function [4].

In recent years, a simple, neck-mounted device has been developed (Swallowscope, GOKURI®︎, PLIMES Inc., Tsukuba, Japan). The GOKURI device uses a condenser microphone to capture swallowing sounds from the skin on the neck and displays the number of swallows in real time. The sounds of swallowing are classified as follows: sound I: the sound made when the epiglottis closes, sound II: when the food mass passes through, and sound III: when the epiglottis opens. By using this device in RSST, it becomes possible to easily obtain the number of swallows in a certain period and also evaluate the duration of each swallow called swallowing time, from the beginning of sound I to the end of sound III, by analyzing the waveform of the swallowing sounds offline, therefore it has the potential to detect the declined swallowing function at an early phase [5,6]. We have already conducted the RSST using GOKURI on 20 elderly individuals who consumed a regular diet and had no history of aspiration pneumonia, reporting that seven individuals (35%) exhibited declining swallowing function [7].

Four stages of decline in oral function in elderly people have been recently proposed: pre-frail, orally frail, oral hypofunction, and oral dysfunction [8]. The criteria for oral dysfunction include dry mouth, decreased tongue pressure, decreased chewing ability, and decreased swallowing function. Interventions for oral dysfunction involve tongue exercises, oral care, rehabilitation, and acupuncture treatment [9-11]. Acupuncture treatment to the acupoints of the lower leg, namely, ST36, located on the front of the lower leg, four fingerbreadths below the patella on the lateral edge of the tibia, and KI3, located between the medial malleolus and the Achilles tendon, was reported to shorten the latency of the swallowing reflex [9] and improve pharyngeal retention and aspiration in VF [10]. We have investigated the changes in swallowing function before and after the acupuncture treatment using the RSST with GOKURI and reported a short-term improvement in swallowing function [7]. Furthermore, analyzing the swallowing sounds obtained by GOKURI, there was no change in the swallowing time before and after the treatment, but a shortening in the interval between swallows was observed [7].

Acupuncture treatment, however, is difficult to perform by oneself at home. An alternative to acupuncture is a non-invasive press needle (PYONEX ZERO®︎, Seirin, Shizuoka, Japan). It has a stainless-steel contact point (press needle) fixed to the adhesive tape and can be attached to acupuncture points on the skin without insertion. It is a commercially available device and can be used safely and easily. If this press needle can achieve the same effect as acupuncture treatment, it will be useful for self-care at home. This study aims to identify factors contributing to declining swallowing function in elderly individuals and assess the effectiveness of non-invasive press needles as a self-care intervention.

## Materials and methods

This study is a prospective study. Twenty-seven individuals aged 65 and over (older adult group) and 28 individuals aged 18 to 64 (younger age group) have participated at Tsukuba University of Technology, Japan. All participants had no history of central nervous system diseases such as cerebrovascular disease, Parkinson's disease, or other diseases that directly cause dysphagia, and did not have any difficulty in swallowing when eating normal food. They also were able to fully understand the instructions given during the examination. This study was approved by the Ethics Committee of the Center for Integrative Medicine, Tsukuba University of Technology (Notification No. 202104, 2021) and has been registered with the University Medical Information Network (UMIN) Clinical Trials Registry (UMIN000047983).

The examination was conducted in a sitting position. First, the amount of moisture in the mouth was measured using an oral moisture meter (Mucus®︎, Life, Japan). The measurement was taken by pressing the sensor vertically against the center of the tongue, about 10 mm from the tip of the tongue, and applying a constant measurement pressure three times in succession, with the average value used for evaluation. If the value is 29.6 or higher, the oral moisture level is assessed as normal, 28.0 to 29.5 is borderline, and if it is less than 28.0, it is considered oral dryness.

Next, we measured tongue pressure using a tongue pressure measuring device (TPM-02E, JMS, Hiroshima, Japan). During measurement, we asked the subject to put the tongue pressure probe on the balloon into their mouth and instructed them to press it as hard as possible between the tongue dorsum and the palate.

**Figure 1:**
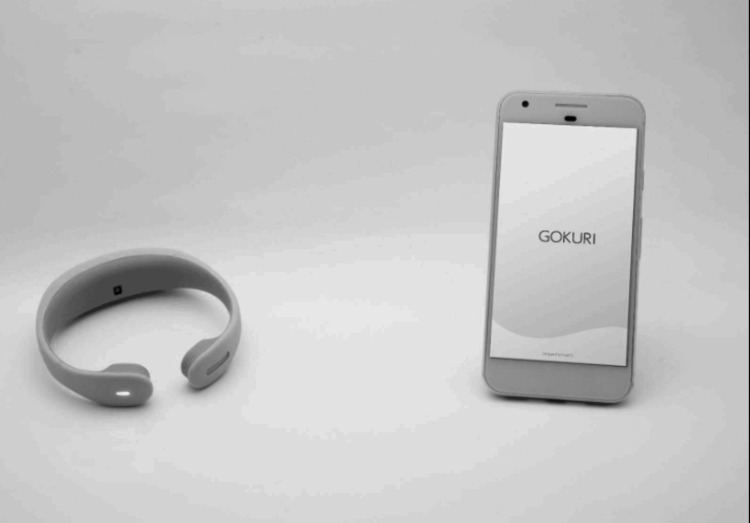
Swallowscope. A piezoelectric-type contact microphone (frequency range: 200 Hz-8 kHz) captured the swallow sounds. A small electret condenser-type microphone is equipped at the tip of the apparatus. The swallowing sound can be analyzed using a smartphone. A wearable, non-wired neckband detects swallow sounds and neck angle changes. The images are already authorized. Credit: Figure provided by PLIMES Inc. Permission to reproduce this image has been obtained.

After these examinations, the GOKURI device was attached to the subject's neck, and the RSST was performed. The examiner gave the instruction "Please swallow as many times as possible," and the number of swallows in 30 minutes was counted by the GOKURI (Figure 1). If the number of swallows was less than three times or if it took more than 10 seconds for the first swallow, it was considered to be a declined swallowing function and was targeted for intervention.

**Figure 2:**
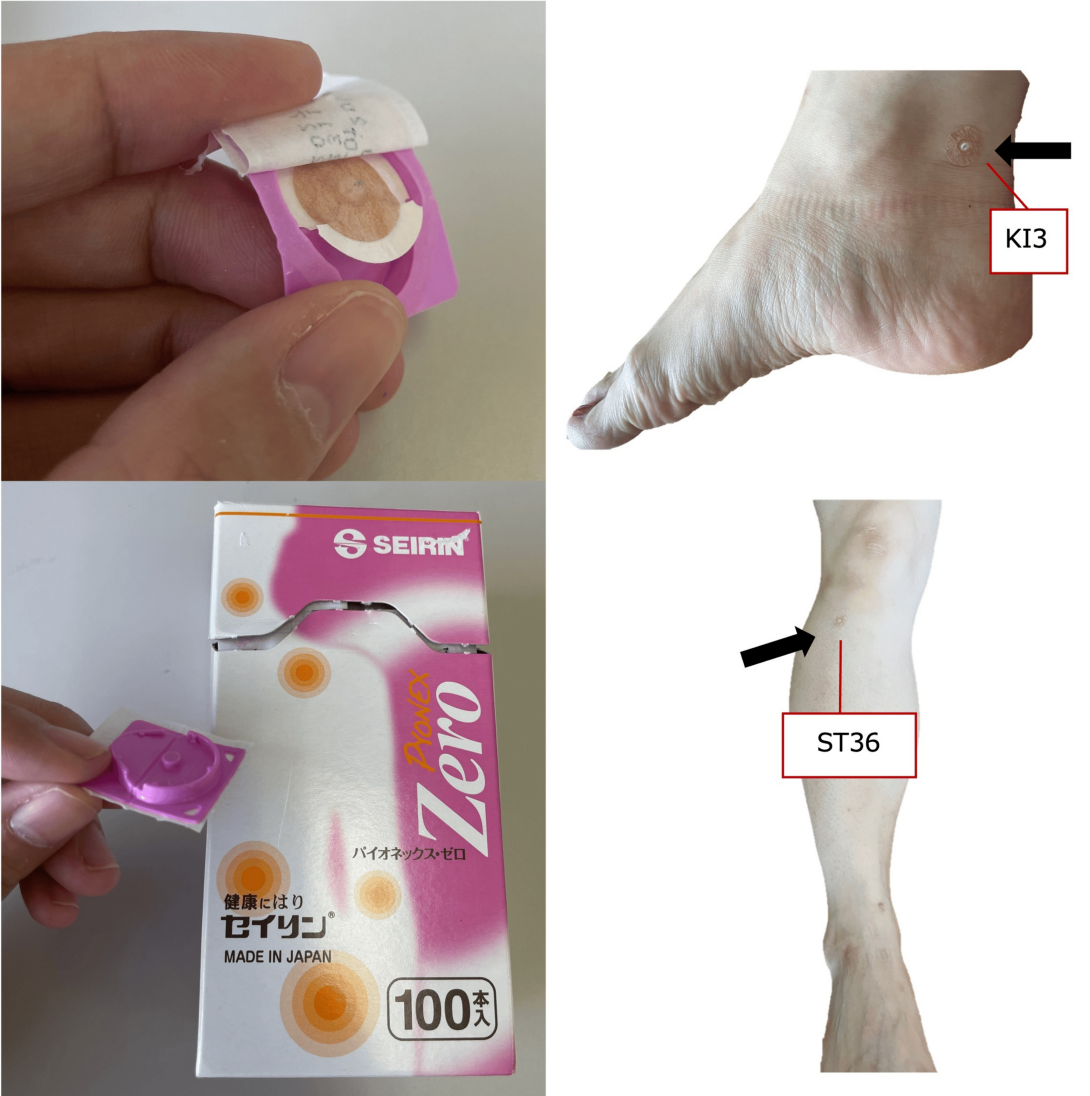
Contactor with tape (left and top) and application to acupuncture points (top right: KI3, bottom right: ST36). Credit: This figure was created by the author.

The intervention involved applying press needles bilaterally to ST36 and KI3 on the legs and providing guidance on how to replace the press needles (Figure 2). The subjects themselves replaced the press needle once a day, and after one week, the amount of moisture in the mouth, tongue pressure, and the RSST were measured again. The subjects received an explanation about self-care from the acupuncturist on the first day of the intervention, but they replaced themselves for the following week.

The Mann-Whitney U test was used to compare the ages of the groups and the chi-square test to the genders. The unpaired t-test was used to compare the oral moisture content, tongue pressure, and RSST between the older adult group and the younger age group. Correlation analysis was also used to examine the relationship between oral moisture content, tongue pressure, and RSST values. The Wilcoxon single-ranked test was used to compare the data before and after the intervention. The significance level was set at less than 5%. In addition, from the data on swallowing sounds obtained by GOKURI, the average swallowing time was measured [5], and the changes before and after the intervention were examined in each group using t-tests. All tests were conducted using IBM SPSS Statistics for Windows, Version 27 (Released 2020; IBM Corp., Armonk, New York, United States).

## Results

Examination of factors contributing to declining swallowing function in terms of oral function

Two participants in the older adult group and one in the younger age group declined to cooperate with the study, and one in the adult group dropped out due to the schedule. The final analysis included 24 participants in the older adult group and 27 in the younger age group (Table 1).

**Table 1:** Characteristics of older age group and younger age group. The asterisk (*) indicates the results of the tongue pressure between the two groups using the unpaired T-test. There was a significant difference in tongue pressure between the older adult and the younger age groups (p=0.049).

	Older adult group	Younger age group		p-value
n	24		27	-
Male/female n (%)	10/14 (41.7%/58.3%)		15/12 (55.6%/44.4%)	0.322
Mean age (years), mean±SD	76.2±7.4		41.8±11.7	<0.001
Tongue pressure (kPa), mean±SD	33.4±7.1		38.4±9.8	0.049*
Saliva (mean±SD)	28.4±5.0		28.1±3.7	0.806
RSST (mean±SD)	3.8±2.1		4.8±2.5	0.161

There was no significant difference in the oral moisture value between the two groups, with an average of 28.4±5.0 in the older adult group and 28.1±3.7 in the younger age group. The mean tongue pressure was 33.4±7.1 kPa in the older adult group and 38.4±9.8 kPa in the younger age group, showing significantly stronger pressure in the younger age group (Table 1, p=0.049).

In the RSST, 7 (29.2%) of the 24 participants in the older adult group and 4 (14.8%) of the 27 in the younger age group showed declined swallowing function. The average tongue pressure of the seven participants in the older adult group was 36.3±4.8 kPa, while the mean tongue pressure of the four participants in the younger age group was 26.9±4.2 kPa, which was lower than the older adult group. Pearson's correlation analysis between two groups was conducted; in the younger age group, a positive correlation was observed between RSST and tongue pressure (r=0.406, p=0.036), while no correlation was observed in the older adult group (r=0.014, p=0.949).

Next, we compared the seven participants with declined swallowing function and the 17 with normal swallowing function in the older adult group; we found no significant differences in oral moisture content, tongue pressure, or swallowing time between the two groups (Table 2).

**Table 2:** Comparison of older adult group with and without impaired swallowing function before self-care patch intervention. There was no significant difference in any of the items (p>0.05). RSST: repetitive saliva swallowing test

	< RSST 3 times	> RSST 3 times	p-value
n	7	17	-
Tongue pressure (mean±SD)	36.3±4.8	32.1±7.6	0.208
Saliva (mean±SD)	26.6±3.5	29.2±5.4	0.262
Swallowing sound (mean±SD)	0.90±0.1	0.89±0.1	0.878

Effects of self-care using a press needle on declining swallowing function

We carried out the self-care intervention with the press needle for 11 participants (seven in the older adult and four in the younger age group) who exhibited impaired swallowing function. The average RSST of the seven participants in the older adult group before the self-care intervention was 1.4±0.8, but after one week there was a significant improvement with an average of 2.9±1.3 (t-test; p=0.047). On the other hand, no significant change was found in the four participants in the younger age group (t-test; p>0.05). No findings for the other items were observed before and after the intervention significantly in both groups (Table 3). In addition, all participants could replace the press needle themselves during the intervention. No side effects were mentioned.

**Table 3:** Comparison of pre- and post-interventions in participants showing with decreased swallowing functions. RSST counts were significantly greater after the self-care treatment in the older adult group (p*=*0.047). RSST: repetitive saliva swallowing test

	Older adult group (n=7)				Younger age group (n=4)			
	pre		post	p-value	pre	post		p-value
Tongue pressure (mean±SD)	36.3±4.8	41.2±5.8		0.128	26.9±4.2		26.6±4.8	1.000
Saliva (mean±SD)	26.6±3.5	28.3±3.8		0.063	28.9±5.4		28.5±1.5	0.715
RSST (mean±SD)	1.4±0.8	2.9±1.3		0.047*	1.0±0.7		1.7±0.4	0.257

Analyzing swallowing time using GOKURI

We analyzed the swallowing time from the swallowing sounds of 22 participants in the older adult group and 26 in the younger age group, excluding three cases (two in the older adult and one in the younger age group) that were difficult to analyze the swallowing time. The average swallowing time of a total of 92 swallows in the older adult group was 0.90±0.14 seconds, and 121 swallows in the younger age group were 0.78±0.14 seconds (t-test; p=0.000).

The average swallowing time before and after the intervention for the five participants with impaired swallowing function in the older adult group was 0.87±0.11 seconds before the intervention and 0.92±0.07 seconds after the intervention (t-test; p>0.05), while the average before intervention in the younger age group was 0.95±0.10 seconds, and 1.00±0.11 seconds after the intervention (t-test; p>0.05). From these results, there was no significant change in the swallowing time before and after the intervention in either group. It should be noted that the RSST value of the three cases in which it was difficult to analyze the swallowing sounds was all less than three.

## Discussion

The main results were as follows: 1) in the comparison between the two groups, the tongue pressure was significantly lower in the older adult group; 2) 29.2% in the older adult group and 14.8% in the younger age group showed declined swallowing function; 3) the tongue pressure of the younger age group in the declined swallowing function was low; 4) after one week of self-care intervention, the older adult group showed significant improvement in RSST.

In this study, we found that approximately 30% of asymptomatic elderly people without dysphagia had impaired swallowing function during the RSST. This result is the same as that of our previous study [7]. Regarding the factors causing a decline in swallowing function, there was no significant difference in intraoral moisture content between the older adult and younger age group who did not present with dysphagia. It was thought that there was no clear relationship between the decline in swallowing function and oral dryness in the asymptomatic elderly. On the other hand, the findings showed that tongue pressure and swallowing time in the older adult group were significantly lower and longer respectively, than those in the younger age group, but there was no significant difference observed between those with and without dysphagia in the older adult group. Therefore, it was thought that the decrease in tongue pressure and the prolongation of swallowing time were general characteristics of elderly people. In a previous study that used a balloon-type tongue pressure measuring device, it was reported that the maximum tongue pressure peaked at 41.9±9.9 kPa in people in their 30s and did not change significantly until their 50s but then decreased to 37.6±8.8 kPa in people in their 60s and 31.9±8.9 kPa in people in their 70s [12]. The swallowing time obtained from the swallowing sounds is generally estimated to be approximately 0.8 seconds [13], and although prolonged swallowing time has been reported in patients with dysphagia [5], however, there have been no previous findings in elderly people without dysphagia. This study showed that swallowing time is also prolonged in the elderly without dysphagia.

In the four participants in the younger age group who were found to have impaired swallowing function, the average tongue pressure was 26.9±4.2 kPa, which was lower than the average in the adult group and this result did not change after the intervention. In addition, a significant positive correlation between the RSST and tongue pressure was observed in the younger age group, but not in the older adult group. Recently, a relation between oral frailty and tongue pressure has been pointed out [14]. A large-scale cohort study of 2,011 elderly people found a significant association between tongue pressure of less than 30 kPa and physical frailty [15]. Another study reported a significant positive correlation between tongue pressure and grip strength [16]. Based on these reports, the participants who showed impaired swallowing function in the younger age group in this study may have frailty. Chen et al. divided 94 elderly participants without dysphagia into sarcopenia and non-sarcopenia groups and evaluated their swallowing function. They reported that in the elderly with sarcopenia, swallowing function potentially declined, but no difference was found in tongue pressure between the sarcopenia and non-sarcopenia groups [3]. In our study, no significant correlation was found between the RSST and tongue pressure in the older adult group, suggesting that the decline in swallowing function in the older adult group may not be related to the tongue pressure, and the mechanism of dysphagia in the older adult group is different from that in the younger age group. This may be reflected in the difference in the effect of the intervention between the two groups.

In this study, the older adult group showed significant improvement in the RSST after one week of self-care intervention with a press needle applied to the acupuncture points of the bilateral ST36 and KI3 on the lower leg. This is the first report of the use of the press needle for the treatment of dysphagia. Acupuncture treatment for ST36 and KI3 has been reported to improve swallowing function by shortening the latency of the swallowing reflex and reducing the incidence of aspiration in VF [10]. Akamatsu et al. [17] reported that 12 patients after a stroke showed a reduction in swallowing reflex latency after four weeks of transcutaneous electrical stimulation (TENS) treatment in the same area. Someya and Akama [18] investigated the mechanism underlying the effect of acupuncture at ST36 and KI3 on swallowing function using functional MRI in 12 healthy subjects. The results showed significant changes in the cluster coefficients in the right anterior cingulate, left nucleus accumbens, and cerebellum, suggesting that acupuncture at these points adjusts the brain function involved in swallowing. In this study, the analysis of swallowing sounds using GOKURI showed no change in the swallowing time before and after the intervention, which was the same result as in our previous research on acupuncture treatment [7]. Furthermore, there was no change in tongue pressure or oral moisture. Therefore, the press needle was considered not to be acting on oral function but on the central function.

This study had several limitations. First, all participants who were found to have impaired swallowing function were targeted for self-care intervention without a control group. Because there is no control group, the natural course of events and placebo cannot be ruled out. It will be necessary to conduct randomized controlled trials in the future. We focused on tongue pressure in this evaluation, but there are various methods for evaluating sarcopenia [19]. In the future, it will be necessary to consider other methods of evaluation. In addition, the evaluation of swallowing sounds in this study was limited to the temporal evaluation of the swallowing number and duration. A future issue is to conduct a qualitative analysis. In addition, we focused on tongue pressure in this evaluation, but there are various methods for evaluating sarcopenia. In the future, it will be necessary to consider other methods of evaluation. Next, the sample size was small, and there was selection bias, so the statistical power may have been limited. The number of cases needs to be increased, and further study is needed. Finally, this study only examined short-term effects. Long-term observational studies are needed to clarify the extent to which the effects of self-care persist.

## Conclusions

Even in elderly individuals without a history of swallowing-related diseases, about 30% showed a decline in function. However, applying self-care contact patches with tape to both the ST36 and KI3 for one week led to short-term improvement in these cases. It is possible that this could be used as self-care for the older adult group with reduced swallowing function in the future. In addition, the fact that there were no changes in oral moisture content, tongue pressure, or swallowing time suggests that it may be acting on other factors related to reduced swallowing function. It would be useful if caregivers or family members could provide the same care to elderly people receiving home care and contribute to improving their swallowing function.
